# Effects of Casein-Derived Peptide Met-Lys-Pro on Systolic and Diastolic Blood Pressure: A Randomized, Double-Blind, Placebo-Controlled, Parallel-Group Study

**DOI:** 10.3390/nu16172975

**Published:** 2024-09-03

**Authors:** Soichiro Sato, Daisuke Ochi, Kazumi Nabeshima, Ryo Sakiyama, Yuki Somoto, Manabu Nakano, Miyuki Tanaka, Masahiko Nakamura

**Affiliations:** 1 Innovative Research Institute, R&D Division, Morinaga Milk Industry Co., Ltd., 5-1-83, Higashihara, Zama 252-8583, Japan; 2 Department of Neurosurgery, Matsumoto City Hospital, 4417-180, Hata, Matsumoto 390-1401, Japan

**Keywords:** casein, peptide, MKP, blood pressure, hypertension

## Abstract

Hypertension is defined as a systolic blood pressure (SBP) of over 140 mmHg or diastolic blood pressure (DBP) of over 90 mmHg. Hypertension is widely known to be a factor affecting human health, so its prevention is considered important. We investigated the effect of casein-derived tripeptide Met-Lys-Pro (MKP) on blood pressure in a randomized, placebo-controlled, parallel-group study. Participants were healthy adults with SBP between 120 and 139 mmHg, and/or DBP between 80 and 89 mmHg. A total of 121 participants were randomly assigned to the MKP group or placebo group. Participants received either a test powder containing 100 μg of MKP or a placebo powder without MKP for 12 weeks. As a result, SBP and DBP were significantly lower in the MKP group than in the placebo group. No adverse events associated with the MKP intake were observed. This study showed that MKP has a beneficial effect on lowering blood pressure in healthy adults with high-normal and elevated blood pressure and can be safely used for continuous intake.

## 1. Introduction

Hypertension is defined as systolic blood pressure (SBP) of over 140 mmHg or diastolic blood pressure (DBP) of over 90 mmHg according to the WHO and the Japanese Society of Hypertension Guidelines for the Management of Hypertension (JSH2019) [1]. In 2019, more than 1.2 billion people had hypertension all over the world, which was about double the number in 1990 [2]. The WHO reports that hypertension is associated with the risk of kidney disease, heart disease, and stroke [3]. Hypertension is considered the largest risk factor for disease burden, which is quantified by indicators such as morbidity and economic losses in addition to disease-related mortality [4]. Therefore, hypertension could be a significant risk factor for our health. Although the modulation of blood pressure is important, it is estimated that more than 40% of hypertensive individuals are not even diagnosed [2]. Thus, there are limitations in the prevention or treatment of hypertension. Following these current situations, the modulation of blood pressure through daily dietary foods or lifestyle habits may be useful for maintaining good health. Also, there are some conditions other than hypertension according to SBP and DBP ranges. High-normal blood pressure is defined as a SBP between 120 and 129 mmHg and a DBP under 80 mmHg. Elevated blood pressure is defined as a SBP between 130 and 139 mmHg, and/or a DBP between 80 and 89 mmHg, according to the guideline JSH2019 [1]. It has been reported that individuals with high-normal and elevated blood pressure who do not develop hypertension have a lower risk of cardiovascular morbidity and mortality than those with hypertension [5]. This report suggests that maintaining blood pressure within the healthy range could be important.

The effectiveness of nutraceutical and functional foods in regulating blood pressure has been reported [6]. For example, milk consumption has the potential to reduce the risk of hypertension [7]. Milk is rich in calcium, vitamin A, vitamin B, and other nutrients, each of which has been reported to modulate blood pressure [8,9,10]. In addition, milk-derived peptides, which are enzymatically produced from proteins, have attracted attention for their diverse physiological effects [11]. One of the beneficial effects of milk-derived peptides is the regulation of blood pressure [12]. The casein-derived tripeptide Met-Lys-Pro (MKP) was found to have high angiotensin I-converting enzyme (ACE) inhibitory activity, which is involved in elevating blood pressure [13]. MKP was also reported to inhibit ACE activity highly compared with food-derived peptides [14]. The ingestion of MKP was shown to lower SBP according to an in vivo study [14]. Furthermore, a 12-week intake of 100 µg of MKP significantly reduced SBP in a clinical study [15]. A significant reduction in SBP was also observed in the subgroup analysis of participants with elevated blood pressure (SBP; 130–139 mmHg, DBP; 85–89 mmHg). Therefore, we focused on the effect of casein-derived tripeptide MKP on maintaining blood pressure in the healthy range and elucidated the lowering effect on SBP and DBP in participants with high-normal and elevated blood pressure in this study.

## 2. Materials and Methods

### 2.1. Study Design and Research Ethics

A randomized, parallel-group, placebo-controlled study was conducted in Matsumoto (Nagano, Japan) from July 2023 to March 2024. This study was approved by the Ethical Review Committee of Matsumoto City Hospital (approval code: 04-5, approval date: 2 August 2022) and registered in UMIN-CTR (UMIN000052102). Before this study was conducted, its purpose and procedures and the rights of the participants were fully explained to the participants, and consent was obtained. This study was conducted in accordance with the Declaration of Helsinki (Fortaleza, revised in 2013) and the Ethical Guidelines for Life Sciences and Medical Research Involving Human Subjects (Ministry of Education, Culture, Sports, Science and Technology, Ministry of Health, Labour and Welfare, and Ministry of Economy, Trade and Industry Notification No. 1, 2021).

Participants were selected from those who provided consent according to the following inclusion and exclusion criteria by the study investigators.

Inclusion criteria: (1) aged 20 to 74 years, (2) SBP less than 140 mmHg and DBP less than 90 mmHg [1], and (3) SBP greater than 120 mmHg or DBP greater than 80 mmHg.

Exclusion criteria: (1) those who were undergoing continuous medical treatment, (2) those who regularly used food for specified health uses, functional foods, and health foods that affect this study, (3) those who smoked 20 cigarettes or more per day, (4) those who drank alcohol polydipsia (pure alcohol equivalent more than 60 g/day), (5) those whose BMIs were >=30 kg/m^2^, (6) those who were under treatment or had a history of disorders such as the heart, liver, kidney, digestive organs, etc., (7) those who had serious allergies to medicines and foods, (8) those who were pregnant, planning to become pregnant, or lactating, (9) those who participated in clinical trials of other drugs or foods within one month, or who planned to do just after giving informed consent, and (10) those who were judged inappropriate to this trial by the principal investigator.

No protocol changes were implemented after consent was obtained.

### 2.2. Intervention

The MKP-containing foods were made from commercially available casein hydrolysate “MKP Hydrolyzed Casein Protein” containing 100 µg of MKP. The placebo foods were without Morinaga Peptide MKP substituted with dextrin. The test foods were provided by Morinaga Milk Industry Co., Ltd. (Kanagawa, Japan). An independent investigator confirmed the indistinguishability of the taste, smell, and appearance of both foods before and at the end of this study. Participants consumed one sachet once daily for 12 weeks with a drink.

Participants were asked to refrain from using pharmaceuticals or consuming healthy foods, functional foods, or dietary supplements that could affect blood pressure during this study. If they ingested any prohibited foods, they recorded them in the participant diary.

### 2.3. Measurement

The time points of measurement were at baseline, 4, 8, and 12 weeks of intake, and 2 weeks after intake. The primary outcome was blood pressure, and SBP and DBP were measured using a sphygmomanometer (HEM-907; Omron Healthcare, Kyoto, Japan) based on the guideline JSH2019 [1]. Prior to the measurements, participants were asked not to consume alcohol before the test day; not to consume caffeine, smoke, or take a bath on the test day until after the measurement; to defecate more than 1 h before the measurement; and to eat meals more than 2 h before the measurement.

Blood pressure was measured by the medical staff after resting for more than 5 min and in a seated position with arms supported at the heart level. Measurements were taken at 1 min intervals and continued until SBP was within 5 mmHg between measurements. The measurement was performed up to 4 times. The average of the last two measurements was used for the analysis.

### 2.4. Diary Survey

Participants recorded daily in the participant diary their intake of test food, sleep status, stress, dietary intake, physical activity, alcohol consumption, health status, hospital visits, and the intake of pharmaceuticals and functional foods. They also completed a dietary questionnaire using the FFQ NEXT ver. 1.0 (Kenpakusha, Tokyo, Japan) at baseline and at Week 12. The nutrient intake was calculated using the software EiyoPlus ver. 1.1 (Kenpakusha, Tokyo, Japan).

The participants who were considered low compliance were excluded from the Per Protocol Set (PPS). Low compliance was defined as the following: those who consumed less than 75% of the test foods, those who had significant lifestyle changes, and those who had inappropriate physical conditions or environments affecting blood pressure, such as injury or irregular sleeping conditions.

### 2.5. Safety Assessment

Adverse events were identified from participant diaries. The severity of adverse events was assessed by the study investigators according to the NCI-CTCAE Version 5.0, Common Terminology Criteria for Adverse Events Ver. 5.0 Japanese translation JCOG version. The association between adverse events and the intake of the test food was judged by the principal investigator.

### 2.6. Sample Size

Based on previous studies [15,16], assuming a between-group difference in SBP and DBP of 3.0 mmHg and a standard deviation of 7.0 mmHg with a significance level of 0.05 and power of 0.80, the required number of participants was calculated to be 174. The number of total cases was 200 considering a dropout rate of approximately 10%.

### 2.7. Randomization

Test foods were allocated 1:1 by the independent assignment manager to a computer-generated allocation table using a block substitution method with block size 4. The allocation manager ensured allocation concealment by randomly assigning participants to test food numbers using computer-generated random numbers. The allocation manager kept the allocation list until the end of this study. Until data were fixed, the allocation table and block sizes were locked to both the participants and the investigators.

### 2.8. Statistical Analysis

The population for efficacy analysis was the Per Protocol Set (PPS). Baseline characteristics were compared by Fisher’s exact test for categorical variables and Student’s *t*-test for continuous variables. For the primary outcome, the effect of MKP was analyzed using analysis of covariance (ANCOVA) with baseline values as covariates. Intragroup comparisons of baseline and at each time point were performed using a paired *t*-test. The change from baseline values was also calculated as an exploratory assessment and compared between groups with an unpaired *t*-test. For the multiplicity of measurement time points, the closed testing procedure was used [17]: tests were performed at 12, 8, and 4 weeks in that order, with the next time point tested only if significant. As an exploratory assessment, ANCOVA including the interaction between MKP and age, sex, or menopause was conducted. Test food intake rates were compared between groups by the Wilcoxon rank sum test. The number and incidence of adverse events and side effects were calculated for each group. The incidence of adverse events was compared between groups using Fisher’s exact test. All statistical analyses were performed using IBM^®^ SPSS ver. 29.0 (IBM Japan, Ltd., Tokyo, Japan). All tests were two-tailed and *p* < 0.05 was considered statistically significant.

## 3. Results

### 3.1. Participants

**Figure 1:**
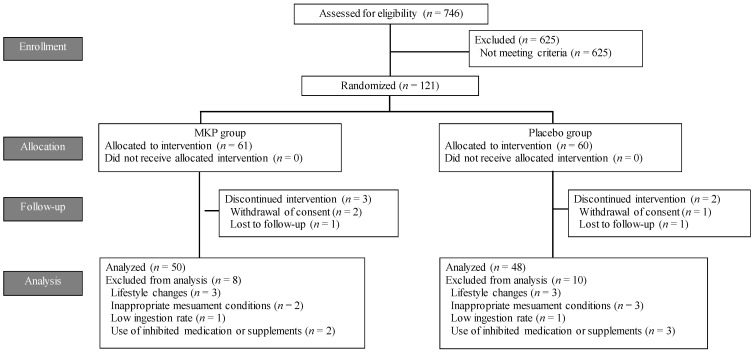
Study flow chart.

The study flow chart is shown in Figure 1. During recruitment (August 2023–September 2023), 746 participants were assessed for eligibility. A total of 635 participants were excluded due to the failure to meet inclusion criteria, their present illness and medical history (such as liver disease, cardiovascular disease, renal disease, diabetes, and dyslipidemia), regular intake of supplements or foods that claimed the lowering effect of blood pressure, or other exclusion criteria. Finally, 121 participants were enrolled and randomly assigned. Participants were confirmed not to be sick by the principal investigator as a doctor. The safety analysis set included 119 participants who consumed the test food at least once, except for 2 participants who were lost to follow-up. A total of 5 participants dropped out due to the withdrawal of consent and lost to follow-up, and 58 participants in the MKP group and 58 participants in the placebo group completed this study. A total of 18 participants (6 participants for lifestyle change, 5 participants for inappropriate measurement environment, 2 participants for low intake of test foods, and 5 participants for use of inhibited medicines or supplements) were excluded as low compliance, so 98 participants (MKP group; *n* = 50, placebo group; *n* = 48) were included in the PPS analysis population. The intake rate of the test food did not differ between the two groups (MKP group; 96.8 ± 4.3%, placebo group; 97.4 ± 4.2%, *p* > 0.1).

### 3.2. Background

The backgrounds of the participants are shown in Table 1. There were no significant differences in background information between the two groups for both Intention to Treat (ITT) and PPS. Lifestyle habits that affect blood pressure, such as smoking and frequency of alcohol consumption, also did not differ between the two groups.

Table 2 also shows the nutrient intake surveys (energy, protein, fat, carbohydrate, fiber, sodium, and potassium) using the FFQ at baseline and Week 12. There were no significant differences in nutrient intake between the two groups at baseline and at Week 12. There were also no significant intragroup differences in nutritional intake from baseline to Week 12. No changes were found in the diary-based exercise habits over the study period in the PPS population.

### 3.3. Effect of MKP Intake on Blood Pressure

**Figure 2:**
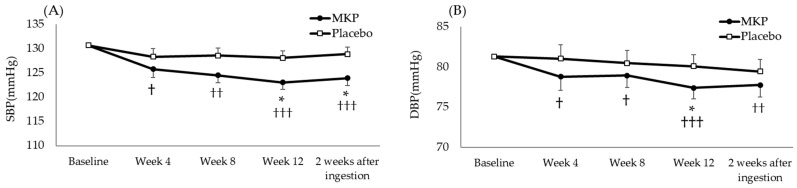
Blood pressure changes over time in each group (**A**) SBP (**B**) DBP (MKP group; *n* = 50, Placebo group; *n* = 48). Values are the least square means ± standard error. Significant difference between the MKP and Placebo group determined by ANCOVA adjusted for the baseline (*; *p* < 0.05). Significant intragroup differences from baseline determined by the paired *t*-test (†; *p* < 0.05, ††; *p* < 0.01, †††; *p* < 0.001).

Figure 2 shows the changes in SBP and DBP from baseline to Week 12 and 2 weeks after ingestion. Two participants in the MKP group at Week 4 and at 2 weeks after ingestion and two participants in the placebo group at Week 8 were missing data due to not performing the measurement. SBP (ls mean ± SE) was 123.0 ± 1.4 mmHg in the MKP group and 128.1 ± 1.4 mmHg in the placebo group at Week 12, which was significantly lower in the MKP group (ANCOVA; *p* < 0.05). SBP was also significantly lower in the MKP group at Weeks 4 to 12 and 2 weeks after ingestion compared with baseline. DBP (ls mean ± SE) was 77.4 ± 0.9 mmHg in the MKP group and 80.1 ± 0.9 mmHg in the placebo group at Week 12, which was significantly lower in the MKP group (ANCOVA; *p* < 0.05). DBP was also significantly lower in the MKP group at Weeks 4 to 12 and 2 weeks after ingestion compared with baseline. The change from baseline to each measurement time point was also analyzed in an exploratory analysis. The change from baseline to Week 12 for SBP (average ± SD) was −7.6 ± 10.2 mmHg in the MKP group and −2.7 ± 10.3 mmHg in the placebo group (unpaired *t*-test; *p* < 0. 05). Similarly, the change from baseline to Week 12 for DBP (average ± SD) was −4.0 ± 6.7 mmHg in the MKP group and −1.1 ± 6.2 mmHg in the placebo group (unpaired *t*-test; *p* < 0.05).

We also assessed whether age, sex, and menopause affect the lowering effect of MKP. The *p*-values of the interaction were greater than 0.15 (SBP; *p* = 0.349, *p* = 0.551, *p* = 0.306, DBP; *p* = 0.191, *p* = 0.509, *p* = 0.731, respectively), and these factors did not affect the effect of MKP.

### 3.4. Safety

There were 261 adverse events during the study period. All adverse events were mild and transient and were not related to the ingestion of test food judged by the principal investigator. There was no significant difference in the incidence of adverse events between the two groups (MKP group; 65.0%, placebo group; 59.3%, *p* > 0.1).

## 4. Discussion

In this study, the 12-week intake of casein-derived tripeptide MKP significantly lowered SBP and DBP in healthy adults with high-normal and elevated blood pressure. Since this study confirmed that there was no change in dietary and exercise habits during the study period based on the participant diary, the blood pressure lowering effect was considered due to the intake of MKP.

In a previous report, MKP lowered SBP and DBP in individuals with elevated blood pressure and grade I hypertension, but there was no statistically significant difference in DBP between the MKP group and placebo group [15]. The previous study included a wider variety of participants in terms of blood pressure. This study included an appropriate number of participants with high-normal and elevated blood pressure. It could confirm the effect of MKP on lowering both SBP and DBP.

MKP is thought to exert its effects on blood pressure through its ACE inhibitory activity. ACE is an enzyme that acts in the renin-angiotensin system and mediates the conversion of angiotensin I to angiotensin II. Angiotensin II is known to cause vasoconstriction [18]. ACE is also thought to be involved in bradykinin inactivation. Bradykinin is known to dilate blood vessels via NO production and antagonism with angiotensin II [19]. Through these mechanisms, inhibition of ACE contributes to the reduction in both SBP and DBP [20].

High blood pressure has been reported to be associated with cardiovascular and other diseases [21]. The results of this study showed that the intake of MKP may be useful for maintaining health. We consider that the effects of MKP were consistent with different food types as the test food was powder in this study and capsule in a previous study [15]. MKP was reported to be rapidly transferred from the intestinal tract into the blood [14] and may exhibit ACE inhibitory activity in vivo [14]. Therefore, the effects of MKP can be applied to various types of food for oral intake. The efficacy of ACE inhibitors for the treatment of hypertension is widely recognized. On the other hand, ACE inhibitors carry a risk of side effects, such as cough and dizziness. In particular, a cough is a common symptom that has been reported to persist for up to 1 month even after discontinuing ACE inhibitor therapy [22]. In this study, no adverse events associated with the intake of MKP were observed after 12 weeks of MKP intake, suggesting that MKP is safe for long-term intake. Blood pressure was also measured in this study at 2 weeks after intake. Although there was a gradual increase in blood pressure, no abrupt changes were observed in the MKP group. In a previous report, in which MKP was ingested at 1000 μg/day for 4 weeks, no adverse events due to MKP intake were observed [23]. These results indicate that MKP is highly safe. It could be important to clarify the functionality of food products that lower high blood pressure without harming health.

There are several limitations in this study. The participants were all healthy adults with high-normal and elevated blood pressure; thus, the effects on children and the sick are unknown. In addition, based on the sample size calculation, it was assumed that this study would be conducted with 200 participants. However, 121 participants met the inclusion criteria and did not meet the exclusion criteria in this study. The difference between the two groups was larger than assumed and the variation in values between participants was small; this might have resulted in significant differences between groups because sufficient power was maintained in this study. Finally, parameters other than blood pressure were not measured. We believe that measuring other parameters related to blood pressure can provide more detailed insight into the effects of MKP.

## 5. Conclusions

This study suggests that MKP intake could be effective in lowering systolic and diastolic blood pressure in healthy adults with high-normal and elevated blood pressure.

## Figures and Tables

**Table 1:** Background of the participants.

	All Participants	ITT Population			PPS Population		
		MKP	Placebo	*p*-Value	MKP	Placebo	*p*-Value
	(*n* = 121)	(*n* = 61)	(*n* = 60)		(*n* = 50)	(*n* = 48)	
Male/Female	67/54	34/27	33/27	1.000	27/23	28/20	0.689
Pre-/Post-menopausal	31/23	15/12	16/11	1.000	12/11	12/8	0.760
Age (years)	54.1 ± 9.7	53.9 ± 9.1	54.4 ± 10.4	0.751	53.7 ± 8.8	55.0 ± 10.7	0.499
BMI (kg/m^2^)	22.3 ± 3.1	22.2 ± 3.2	22.3 ± 3.2	0.912	22.0 ± 3.1	22.2 ± 2.7	0.718
SBP (mmHg)	130.8 ± 5.6	130.7 ± 5.2	130.8 ± 6.0	0.939	130.5 ± 5.4	130.8 ± 5.8	0.776
DBP (mmHg)	81.2 ± 5.3	81.4 ± 4.9	81.0 ± 5.7	0.689	81.8 ± 4.6	80.7 ± 5.8	0.299
Smoker/Non-smoker	6/115	5/56	1/59	0.207	3/47	0/48	0.243

Values are means ± standard deviation. No significant differences were observed between the groups.

**Table 2:** Nutrient intake during the intervention.

		MKP	Placebo
Energy (kcal)	Baseline	1875.2 ± 215.1	1917.2 ± 283.9
	Week 12	1865.6 ± 207.6	1887.3 ± 233
Protein (g)	Baseline	73.1 ± 6.5	75.4 ± 8.9
	Week 12	73.5 ± 6.8	74.5 ± 8.1
Fat (g)	Baseline	57.4 ± 4.3	59.1 ± 8.2
	Week 12	58.2 ± 5.7	58.2 ± 5.0
Carbohydrates (g)	Baseline	265.7 ± 33.0	265.8 ± 33.5
	Week 12	259.5 ± 31.1	262.2 ± 30.8
Fiber (g)	Baseline	14.6 ± 1.4	14.9 ± 1.7
	Week 12	14.6 ± 1.5	14.7 ± 1.5
Sodium (mg)	Baseline	3951.6 ± 465.7	4000.2 ± 517.7
	Week 12	3957.8 ± 429.8	3944.4 ± 440.3
Potassium (mg)	Baseline	2763.7 ± 206.3	2849.6 ± 274.9
	Week 12	2767.6 ± 198.6	2794.0 ± 222.3

Week 0 data were missing for 1 participant in the MKP group (MKP group; Week 0 *n* = 49, Week 12 *n* = 50, Placebo group; Week 0 and 12 *n* = 48). Values are means ± standard deviation. No significant differences were observed between groups and intragroup from Week 0 and Week 12.

## Data Availability

The data presented in this study can be found in this published article.
